# Patient-reported benefit of ReSTOR^® ^multi-focal intraocular lenses after cataract surgery: Results of Principal Component Analysis on clinical trial data

**DOI:** 10.1186/1477-7525-6-10

**Published:** 2008-01-24

**Authors:** Gilles Berdeaux, Muriel Viala, Aude Roborel de Climens, Benoit Arnould

**Affiliations:** 1 Alcon France, Rueil-Malmaison, France; 2 Conservatoire National des Arts et Métiers, Paris, France; 3 Mapi Values, Lyon, France

## Abstract

**Background:**

Restoration of functional distance and near vision independently of additional correction remains a goal for cataract surgery. ReSTOR^®^, a new multi-focal intraocular lens (IOL) addresses this issue with an improvement in both distance and near vision, often without need for glasses. This analysis attempted to discuss the patient-reported benefit of ReSTOR^® ^using a full but organised representation of data.

**Methods:**

Two non-randomised, open-label clinical trials conducted in Europe and the United-States were conducted to compare the efficacy of ReSTOR^® ^to AcrySof^® ^mono-focal IOLs. A total of 710 patients in need of bilateral cataract extraction were included in the pooled study. The TyPE, a patient questionnaire, was fully completed by 672 of them before and after each eye surgery. The TyPE, composed of 67 items measuring overall visual functioning in both conditions (with and without wearing glasses), evaluates limitations, troubles and satisfaction in distance and near vision. A principal component analysis (PCA) of the TyPE questionnaire was performed on pooled data from baseline and post-surgery observations in order to fully represent the change in the TyPE data over time. ReSTOR^® ^and mono-focal groups were used as illustrative variables. The coordinates of the first 2 factors were compared between visits and between IOLs (ReSTOR^® ^vs. mono-focal), using paired t-tests and t-tests, respectively.

**Results:**

The first factor of the PCA explained 55% of the variance and represented 'visual functioning and patient satisfaction'. The second factor explained 6% of the variance and was interpreted as 'independence from glasses'. An overall difference in factorial coordinates in both factors was seen between baseline and the first eye surgery, and between the first and the second eye surgery. No difference between ReSTOR^® ^and mono-focal IOL groups was observed at baseline. After surgery, ReSTOR^® ^treated-patients had higher coordinates on both "visual functioning and satisfaction" and "independence from glasses" factors. Findings observed on the factorial plan were supported by statistical comparisons of factorial coordinates.

**Conclusion:**

Both mono-focal and ReSTOR^®^-implanted patients improved their visual functioning after bilateral cataract surgery. Moreover, ReSTOR^® ^patients reported an additional benefit in independence from glasses as well as in visual functioning and patient satisfaction.

## Background

Cataracts are a clouding of the lens or its surrounding transparent membrane, characterised by a forward light scatter, and therefore stops it from focusing on the retina [1]. Most cataract cases are age-related and may occur in both eyes in a long term perspective. This results in blurred vision and visual disturbances including difficulty in night vision, halos and sensitivity to glare. In 1994, an estimated 38 million people were blind worldwide; 40% of the cases were due to cataracts [2].

There is no effective prevention for cataract today and the only way to treat it is to remove the clouded lens. Most of the time, cataract surgery is performed using phaco-emulsification through a small surgical incision in the Western developed countries [1,3]. The natural lens is then replaced with an artificial intraocular lens (IOL).

Mono-focal IOLs allow either distance or near vision to be corrected, while the other vision has to be corrected by wearing glasses. Trial patients receiving the mono-focal IOL usually chose distance correction and rely on eyeglasses for other distances. To mimic the ability of the natural crystalline lens to focus on near objects, multi-focal IOLs were developed. Multi-focal IOLs provide vision over a range of distances through the provision of two primary lens powers, one power used for distance vision and the other one for near vision. Implantation of multi-focal IOLs after cataract surgery was reported to restore both distance and near vision of patients and to provide them the ability to be free of glasses [4-12].

The AcrySof^® ^ReSTOR^® ^multi-focal IOL is a biconvex single piece and consists of a high refractive index soft acrylic material. Its anterior surface is made of apodized, diffractive concentric rings in the central area distributing the light for a full range of vision [13]. The ReSTOR^® ^lens can be folded prior to insertion allowing placement through an incision smaller than the optic diameter of the lens. The efficacy and safety of ReSTOR^® ^has been reported by numerous papers [14-18].

A number of studies have demonstrated the importance of the patient-reported measures in clinical trials [19]. The TyPE questionnaire, a validated 67-item questionnaire measuring visual functioning relative to multi-focality [5], showed that ReSTOR^® ^was able to significantly improve near vision without glasses after cataract surgery, compared to AcrySof^® ^mono-focal IOLs, allowing the majority of ReSTOR^®^-implanted patients to be free of glasses [15,16]. In contrast with previous studies which used dimension scores of the TyPE questionnaire, the objective of the present study was to fully present the change in patient-reported visual functioning after cataract surgery using the information available in all the individual items of the TyPE questionnaire. This was undertaken using a Principal Component Analysis (PCA) which allows the large amount of data provided by the TyPE questionnaire to be summarised, and thus the main information to be extracted and interpreted. The goal of this analysis was not to psychometrically validate new scores for the TyPE questionnaire, but to provide an alternative way of analysing the richness of the TyPE data with a full but organised representation of its items, and thus to confirm the superiority of ReSTOR^® ^in terms of patient-reported vision benefit.

## Methods

Institutional review board or corresponding ethics committee approvals were obtained for all trials contributing to the current analysis. Written informed consent was obtained and studies conducted. The research followed the tenets of the declaration of Helsinki.

This study reports results from 2 multi-centre open-label non-randomized clinical trials [15,16]. These trials evaluated the safety and efficacy of the new AcrySof^® ^ReSTOR^® ^multi-focal IOL (model MA60D3) compared to the AcrySof^® ^mono-focal IOL (model MA60BM). The similar design of both trials allowed data to be pooled. A total of 710 patients aged 21 and over and in need of cataract surgery were bilaterally implanted with ReSTOR^® ^or mono-focal IOLs after phacoemulsification. All patients included in the study signed an informed consent form.

The eye diagnosed with the most advanced cataract was implanted first. The decision to proceed with bilateral implantation was made by the subject and the investigator, based on the results of the visit following the first surgery (30–60 days). The second implantation was carried out within 90 days of the first one.

The 67-item TyPE questionnaire was completed before and after cataract surgery by patients enrolled in the two multi-centre clinical trials. The TyPE questionnaire was developed as an endpoint to measure visual functioning relative to multi-focality [5,6]. This instrument was administered during this study to rate the patient-reported visual functioning after ReSTOR^® ^and mono-focal implantation following cataract surgery.

The TyPE questionnaire is described in Table 1. It is composed of 67 items organised into 4 sections: section I is related to the frequency of wearing glasses (3 items); section II is related to vision without correction with glasses (30 items about limitations and satisfaction in near and distance vision at night and during the day without glasses); section III is related to vision with correction with glasses (the same 30 items as section II are asked but with glasses); section IV asks global questions (4 items about 'willingness to pay' and 'recent health and happiness').

**Table 1:** Description of the TyPE questionnaire

**Sections**	**Items**	**# of items**	**Range of scale and answer choices**
**Section I: Frequency of wearing glasses**	Frequency of wearing glasses	1	from 1 "always" to 3 "never"
	Frequency of wearing glasses for distance tasks	1	from 0 "none of the time" to 4 "all of the time"
	Frequency of wearing glasses for distance and near tasks	1	from 0 "none of the time" to 4 "all of the time"
**Section II: Vision without glasses**	Self-reported rating of vision without glasses	1	from 1 "the worst" to 10 "the best"
	Trouble with vision during the day without glasses	1	from 0 "no trouble at all" to 4 "major or overwhelming trouble"
	Trouble with vision at night without glasses	1	from 0 "no trouble at all" to 4 "major or overwhelming trouble"
	Satisfaction with overall vision without glasses	1	from 0 "not at all satisfied" to 4 "completely satisfied"
	Satisfaction with vision during the day without glasses	1	from 0 "not at all satisfied" to 4 "completely satisfied"
	Satisfaction with vision at night without glasses	1	from 0 "not at all satisfied" to 4 "completely satisfied"
	Effect of bright light without glasses	1	from 0 "it makes it much better" to 4 "it makes it much worse"
	Trouble with glare and halo without glasses	6	from 0 "no trouble at all" to 4 "major or overwhelming trouble"
	Limitations in distance vision without glasses	7	from 0 "no limitation" to 4 "extreme limitation"
	Limitations in near vision without glasses	5	from 0 "no limitation" to 4 "extreme limitation"
	Limitations in social activities without glasses	5	from 0 "no limitation" to 4 "extreme limitation"
**Section III: Vision with glasses**	Self-reported rating of vision with glasses	1	from 1 "the worst" to 10 "the best"
	Trouble with vision during the day with glasses	1	from 0 "no trouble at all" to 4 "major or overwhelming trouble"
	Trouble with vision at night with glasses	1	from 0 "no trouble at all" to 4 "major or overwhelming trouble"
	Satisfaction with overall vision with glasses	1	from 0 "not at all satisfied" to 4 "completely satisfied"
	Satisfaction with vision during the day with glasses	1	from 0 "not at all satisfied" to 4 "completely satisfied"
	Satisfaction with vision at night with glasses	1	from 0 "not at all satisfied" to 4 "completely satisfied"
	Effect of bright light with glasses	1	from 0 "it makes it much better" to 4 "it makes it much worse"
	Trouble with glare and halo with glasses	6	from 0 "no trouble at all" to 4 "major or overwhelming trouble"
	Limitations in distance vision with glasses	7	from 0 "no limitation" to 4 "extreme limitation"
	Limitations in near vision with glasses	5	from 0 "no limitation" to 4 "extreme limitation"
	Limitations in social activities with glasses	5	from 0 "no limitation" to 4 "extreme limitation"
**Section IV: Global questions**	Willingness to pay	2	from 1 "nothing" to 6 "more than five dollars"
	Recent health and happiness	2	from 1 "the worst" to 10 "the best"

The TyPE items were analysed using PCA, a factorial analysis that reduces the dimensionality of a large number of interrelated variables, while retaining as much as possible the variation present in the data set. The large number of variables is reduced to a conceptually more coherent set of variables, called factors. We focussed on the 60 items of the TyPE which measured patients' visual functioning and their resulting satisfaction with vision. Items 4 to 63 were analysed as active variables of PCA, whereas items 1 to 3 (which are not related to visual functioning or satisfaction but to frequency of wearing glasses) were used as illustrative variables to identify patient sub-groups. Items 64 to 67, measuring different concepts ('willingness to pay' and 'recent health and happiness'), were also used as illustrative but not reported here because they were not considered useful for the purposes of this analysis. This analysis was carried out on the overall population with the 3 time points (one before and two after surgery) pooled together.

PCA is defined as an orthogonal linear transformation that transforms the data to a new coordinate system such that the greatest variance by any projection of the data comes to lie on the first coordinate (called the first principal factor), the second greatest variance on the second coordinate, and so on. The items contributing to the first 2 factors were described using a correlation circle representation. We used the patient coordinates, after rotation, projected on axis 1 and 2, and we calculated average per type of IOL, to compare the impact on vision of each IOL using all the items reported in the TyPE, without any a priori knowledge. Paired t-tests and t-tests were used to compare the factorial coordinates but this should be considered as a multivariate descriptive analysis. In addition, the distribution of the factorial coordinates was depicted per type of IOL by showing the percentages of patients bilaterally implanted at or above factorial 1 and 2 coordinates. The aim of this study was to describe the overall pattern of results rather than test the statistical significance of differences. Because of this, no adjustments were used for multiplicity of tests. Where specific significance tests were used, the threshold for statistical significance was p < 0.05 for each test, with all tests interpreted two sided. The statistical analysis was conducted with the SAS software (SAS Institute; NC), release 9.2, and SPAD software V6.5.

## Results

### Description of the pooled population at baseline

Among the patients included in the clinical trials, 672 patients fully completed the TyPE questionnaire at baseline (pre-operation), 30–60 days after the 1^st ^eye surgery and 120–180 days after the 2^nd ^eye surgery. Out of the 672 patients, 499 received ReSTOR^® ^multi-focal IOLs and 173 were implanted with AcrySof^® ^mono-focal IOLs.

Among the 672 patients of the studied population, 34% were male (Table 2). The mean age of the patients was 69 years, with the ReSTOR^®^-implanted patients being statistically younger than the mono-focal-implanted patients (68.9 versus 70.5 years, with p = 0.02). Before surgery, the mean corrected distance-visual acuity (VA) of the whole population was poor and no difference was seen between ReSTOR^® ^and mono-focal patients (0.39 and 0.50 logMAR units for "distance best corrected VA" and "near chart photopic distance corrected VA at best distance", respectively (Table 2).

**Table 2:** Demographic and visual acuity characteristics of the pooled population at baseline (N = 672)

**Male**	n (%)	229 (34)
**Age (years)**	Mean ± SD	69.1 ± 9.2
**Distance best corrected VA at BL**	Mean ± SD (logMAR units)	0.39 ± 0.21
**Near chart photopic distance corrected VA at best distance at BL**	Mean ± SD (logMAR units)	0.50 ± 0.26

BL: baseline; VA: visual acuity. Scores range from -0.3 (bad vision) to +1.0 (excellent vision) logMAR units.

### Correlation circle of the PCA

The 2 first factors resulting from the PCA accounted for 55% and 6% of the total variance, respectively. As the first factor explained the majority of the variance, this showed that the data are almost uni-dimensional, i.e. many items tend to consistently measure a common underlying concept.

**Figure 1:**
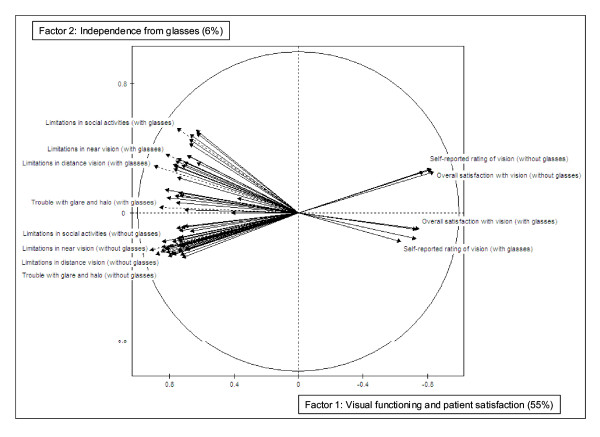
Correlation circle representation of the PCA active variables (TyPE items 4 to 63) considering factors 1 and 2.

A correlation circle representation of the PCA active variables (TyPE items 4 to 63) was drawn to show the items contributing to factors 1 and 2 (Figure 1). This figure is a simple way to represent the 60 by 60 correlation matrix of the TyPE items. Variables close together have a positive correlation, variables in the opposite direction are negatively correlated, and orthogonal variables are independent of each other.

On the horizontal axis, the 1^st ^factor is negatively correlated with *visual limitation *and *visual trouble *items and positively with *satisfaction *items. This shows that the more visually limited the patients, the less satisfied with their vision they are. Inversely, the most satisfied patients are also those who are the least limited. Therefore, these items contribute to define the 1^st ^factor as "visual functioning and patient satisfaction". On the vertical axis the 2^nd ^factor was interpreted to be "independence from glasses." Various difficulties with glasses, vision satisfaction without glasses, and higher ratings of vision without glasses positively correlate with the idea of being independent of glasses. Conversely, difficulties without glasses, vision satisfaction with glasses, and better rating of vision with glasses would reflect more dependence on glasses, i.e. less independence.

### Factorial plan of the PCA

**Figure 2:**
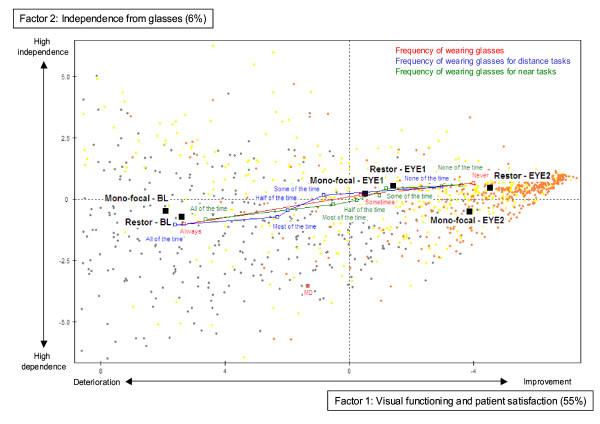
**Location of patients scores in the factorial plan composed by factors 1 and 2**. Grey dots correspond to assessment at baseline (BL), yellow dots to assessment after the 1st eye surgery (EYE1) and orange dots to assessment after the 2nd eye surgery (EYE2).

The factorial plan presented in Figure 2 corresponds to the projection of all patient coordinates according to factors 1 and 2 axes. Most of the variance can be seen on the horizontal axis (55% explained), reflecting the improvement of the whole population in "visual functioning and patient satisfaction" after the 1^st ^eye and after the 2^nd ^eye surgery. Factor 1 showed a difference in factorial coordinates between baseline and after the 1^st ^eye surgery, and a difference between the 1^st ^and 2^nd ^eye surgeries, indicating that the cataract surgery increased the visual functioning and satisfaction of patients as soon as the 1^st ^eye was operated on, and that another improvement was seen when both eyes were operated on. The 2^nd ^most important source of variance, represented on the vertical axis, corresponds to "independence from glasses". Even though this axis explains only 6% of the variance of the TyPE data, the information extracted by this axis is independent from the one extracted by the first axis and therefore discloses new data. The projection of the 3 illustrative variables of frequency of wearing glasses (empty squares) on the 1^st ^factorial plan showed that the improvement of the visual functioning was related to a decrease in frequency of wearing glasses (Figure 2). On Figure 2, black squares correspond to the projection of the treatment groups at baseline, after the 1^st ^and after the 2^nd ^surgery on the factorial plan. They illustrate the overall difference in the factorial coordinates on the "visual functioning and satisfaction" factor, indicating an improvement in both treatment groups after the 1^st ^eye surgery and after the 2^nd ^eye surgery, with the ReSTOR^® ^group tending to show a larger improvement compared to the mono-focal group. The projection of the treatment groups on the second factor axis showed a difference in their factorial coordinates between baseline and the 1^st ^eye surgery, indicating that both groups tended to stop wearing glasses after the 1^st ^eye surgery. After the 2^nd ^eye surgery, ReSTOR^® ^patients remained free from glasses whereas the mono-focal patients again became dependent on glasses.

In order to quantify these results, factorial coordinates of the pooled population were compared between visits using statistical tests (Table 3). Significant improvements from baseline were observed on both factor axes after the 1^st ^and after the 2^nd ^eye surgery, as well as when comparing the improvement from the 1^st ^to the 2^nd ^eye surgery (all p values < 0.0001).

**Table 3:** Factorial coordinates of ReSTOR^® ^and mono-focal mean scores considering factors 1 and 2

		**Mono-focal **(N = 173) Mean ± SD	**ReSTOR**^® ^(N = 499) Mean ± SD	**p values**^†^
**Factor 1: Visual functioning and patient satisfaction**	At baseline	5.91 ± 5.15	5.39 ± 4.52	0.2451
	After 1^st ^eye surgery	-0.51 ± 4.56	-1.41 ± 4.14	**0.0166**
	After 2^nd ^eye surgery	-3.86 ± 2.63	-4.52 ± 2.78	**0.0067**
**Factor 2: Independence from glasses**	At baseline	-0.50 ± 2.53	-0.74 ± 2.41	0.2647
	After 1^st ^eye surgery	0.22 ± 2.03	0.54 ± 1.73	0.0646
	After 2^nd ^eye surgery	-0.52 ± 1.53	0.47 ± 0.86	**<0.0001**

† p values correspond to t-tests.

Differences between ReSTOR^® ^and mono-focal groups were shown by comparison of their factorial coordinates. At baseline, ReSTOR^® ^and mono-focal coordinates were equivalent on the 1^st ^and the 2^nd ^factor axes (p = 0.2451 and p = 0.2647, respectively). After the 1^st ^eye surgery, a great shift was observed on the 1^st ^factor axis in both ReSTOR^® ^and mono-focal groups indicating an improvement in "visual functioning and patient satisfaction" in both groups (Table 3). However, ReSTOR^® ^patients had significantly higher coordinates than mono-focal patients, meaning that ReSTOR^® ^patients improvement was better than the mono-focal group (p = 0.0166). This difference in "visual functioning and patient satisfaction" between ReSTOR^® ^and mono-focal patients' coordinates was confirmed after the 2^nd ^eye surgery (p = 0.0067). On the 2^nd ^factor axis, patients bilaterally implanted with ReSTOR^® ^had also higher coordinates than mono-focal patients after the 2^nd ^eye surgery (p < 0.0001).

**Figure 3:**
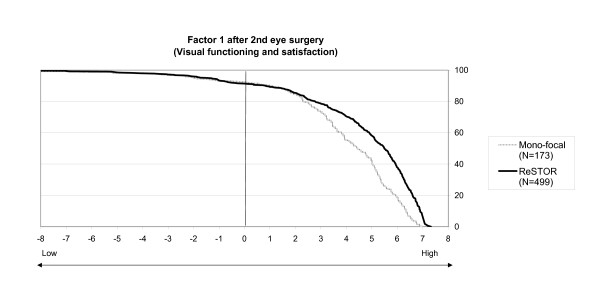
**Visual functioning and satisfaction after the 2^nd ^eye surgery**. Percentages of patients (x axis) who have factorial 1 coordinates at or above values on the y axis, i.e. 80% of patients of mono-focal and ReSTOR^® ^treatment groups have a factorial coordinate ≥ 2.5 (N= 499 for ReSTOR and N= 173 for mono-focal).

**Figure 4:**
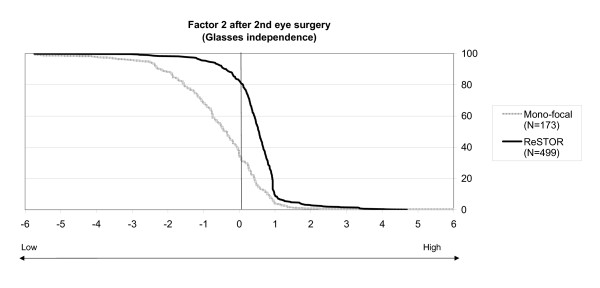
**Glasses independence after the 2^nd ^eye surgery**. Percentages of patients (x axis) who have factorial 2 coordinates at or above values on the y axis, i.e. 50% of patients of ReSTOR^® ^treatment groups have a factorial coordinate ≥ 0.5 (N= 499 for ReSTOR and N= 173 for mono-focal).

The factorial scores were also represented on Figures 3 and 4 that depict the decumulative percentage of bilaterally implanted patients at or above factorial coordinates on factors 1 (Fig. 3) and 2 (Fig. 4). It shows that "Visual functioning and satisfaction" of the ReSTOR^® ^patients was never less than that of monofocal IOL patients and that a difference is noticeable in 80% of the population bilaterally implanted (Figure 3). The results concerning "Glasses independence" were even stronger with a very clear difference between the two curves supporting a better outcome of ReSTOR^® ^(Figure 4).

## Discussion

The objective of this study was to quantify vision benefit following cataract surgery as reported by the patients. A large number of patients in need of cataract surgery and enrolled in 2 multi-centre clinical trials were bilaterally implanted with either AcrySof^® ^ReSTOR^®^, or a mono-focal control IOL. To assess patient-reported visual functioning, patients filled in the 67-item TyPE questionnaire before cataract surgery, after the 1^st ^and after the 2^nd ^eye surgery.

The multiplicity constituting the patient's perspective is reflected by the high number of items in the TyPE questionnaire. Because pre-specification was not possible in this post-hoc analysis, we chose to analyse TyPE data obtained before surgery, after the 1^st ^and after the 2^nd ^eye surgery using PCA. The PCA was carried out on the overall population with the 3 TyPE assessments being analysed together. The analysis taking into account the 3 time points enables dynamic interpretation of visual functioning evolution. Indeed PCA is a powerful method to organise multiple variables that are more or less correlated, and thus reveals new meaningful information. Moreover PCA does not need rigorous distributional assumptions such as normality when it is used as a descriptive tool [20]. This is why PCA is an appealing method to deal with multiplicity issues [21].

Our work did not explore new scoring methods based on classical or modern psychometric theory, such as Rasch Analysis. Revisiting the scoring algorithm of the TyPE could be interesting but goes beyond the scope of our work. Indeed, the original contribution of PCA is that the overall information is summarised and organised without assumptions about the conceptual content of the scale. As TyPE scores were constructed on clinical arguments, it was important to analyse results without assumptions concerning item correlations. The benefit of this method was to reveal two meaningful independent domains of improvement for the patients. The main factor revealed by the PCA was interpreted as "visual functioning and patient satisfaction". This 1^st ^factor accounted for more than half the total variance (55%). The 2^nd ^discriminant factor of change (6% of the total variance) resulting from the PCA was defined as "independence from glasses". This 2^nd ^factor opposes "with glasses" and "without glasses" items. Beyond the confirmation of the benefit of cataract surgery on visual functioning and patient satisfaction, a second interesting direction of improvement independently reported by patients is the free from glasses vision. Despite a structure which the PCA reveals as strongly unidimensional, it is noticeable that the second source of variance in our dataset can so easily be interpreted. The PCA results demonstrated that ReSTOR^® ^and mono-focal patients reported improvement in "visual functioning and satisfaction". However, the visual functioning improvement perceived by ReSTOR^® ^patients was higher than the one with mono-focal IOLs. Moreover, the independence from glasses already reported by multi-focal implanted-patients [4-6,8-10,12] was higher with ReSTOR^® ^than with mono-focal IOLs after the 2^nd ^eye surgery. Indeed, both groups of patients tended to stop wearing glasses after the 1^st ^surgery probably because of the bother caused by their glasses correcting one eye only. ReSTOR^®^-implanted patients were still free from glasses when both eyes were operated, while mono-focal implanted patients were again dependent from glasses after the 2^nd ^eye is operated.

According to the theoretical model of Oliver, patients' satisfaction results from the comparison between their initial expectations and the performance they eventually perceive from their treatment [22]. As a result, the higher expectations are, the more difficult it is to satisfy them. As the decision to be bilaterally implanted with ReSTOR^® ^was made by the patients themselves, they had a high level of expectations. In this context, the high level of independence from glasses reported by patients with bilateral implantation of ReSTOR^® ^supported the value of ReSTOR^® ^on near vision without glasses.

A prospective randomised study previously carried out with patients in need of cataract surgery showed that 60% of them reported discomfort when using glasses for near vision [8]. After cataract surgery, the multi-focal implanted-patients of this study reported a higher level of satisfaction with their near vision than the mono-focal implanted-patients [8]. ReSTOR^® ^results are in accordance with these data; ReSTOR^® ^patients were more satisfied and more easily performed activities requiring near vision without glasses than mono-focal patients [15,16,18]. It should be noticed that Acrysof was chosen since it shares the same platform as Acrysof ReSTOR^®^, therefore minimising the factors that could confound the comparisons between multi-focal and mono-focal IOLs. Consequently, results may not be generalised to other IOLs.

It was also clearly demonstrated that improvement in health-related quality of life (HRQoL) occurred when visual functioning improved following cataract surgery [23]. However, the present study relates only the functional aspects of vision evaluated by the TyPE. A more complete analysis of ReSTOR^® ^patients' HRQoL could be undertaken using validated questionnaires such as the 25-item National Eye Institute Visual Function Questionnaire (NEI-VFQ25) developed to assess the influence of visual disability on HRQoL in patients with chronic eye diseases or low vision [24], or the Quality of Life Impact of Refractive Correction (QIRC) questionnaire developed to quantify the HRQoL of people with refractive correction by spectacles, contact lenses or refractive surgery [25]. Evaluating HRQoL aspects such as psychological, social and emotional well-being in ReSTOR^® ^patients would bring to light further information.

## Conclusion

In summary, these analyses showed a full but organised description of the change in patient-reported visual functioning after cataract surgery. The main factor of "visual functioning and patient satisfaction" resulting from the Principal Component Analysis separates baseline, 1^st ^and 2^nd ^eye surgery TyPE assessments. The 2^nd ^most important source of variance resulting from the PCA and corresponding to "independence from glasses", allowed differentiation between mono-focal and ReSTOR^® ^patients after the 2^nd ^eye surgery. This original representation of the TyPE data confirms that while mono-focal patients did improve their visual functioning and satisfaction after cataract surgery, ReSTOR^® ^patients rated an additional benefit in visual functioning and satisfaction as well as in independence from glasses. The full benefit was reached after the second surgery.

## Abbreviations

HRQoL: Health-Related Quality of Life; IOL: Intraocular lens; PCA: Principal Component Analysis.

## Competing interests

GB is an Alcon employee. This project was funded by an unrestricted grant provided by Alcon France and conducted by MAPI values, Lyon, France.

## Authors' contributions

GB designed the study. MV performed the statistical analyses. MV, BA, ARC and GB analysed the data and prepared the manuscript. ARC wrote the manuscript. All authors read and approved the final manuscript.
